# Voltage-controlled two-dimensional Fresnel diffraction pattern in quantum dot molecules

**DOI:** 10.1038/s41598-024-55204-4

**Published:** 2024-03-09

**Authors:** Hamed Mehrabzadeh, Hamid Khoshdel, Mohammad Mahmoudi, Zahra Amini Sabegh, Saifollah Rasouli

**Affiliations:** 1 Department of Physics, University of Zanjan, University Blvd., Zanjan, 45371-38791 Iran; 2 Department of Physics, Institute for Advanced Studies in Basic Sciences (IASBS), Zanjan, 45137-66731 Iran; 3 Optics Research Center, Institute for Advanced Studies in Basic Sciences (IASBS), Zanjan, 45137-66731 Iran

**Keywords:** Nonlinear optics, Applied optics, Nonlinear optics, Applied optics

## Abstract

This study explores the influence of inter-dot tunneling effects within a quantum dot molecule on the Fresnel diffraction phenomenon. Our findings indicate that the Fresnel diffraction of the output probe Gaussian field can be manipulated by adjusting the inter-dot tunneling parameter’s strength and the characteristics of the coupling field. The inter-dot tunneling effect establishes a closed-loop system, setting conditions for the interference of the applied fields. We specifically examine a Laguerre–Gaussian (LG) coupling field, investigating how its properties-such as strength, value, and sign of the orbital angular momentum (OAM)-impact the Fresnel diffraction of the output probe field. Increasing the inter-dot tunneling parameter and the coupling LG field’s strength allows for control over the spatial distribution of the Fresnel diffraction pattern. Notably, the inter-dot tunneling parameter can disturb the symmetry of the diffraction patterns. Additionally, considering a negative OAM for the coupling LG field transforms the diffraction pattern into its inverse shape. This suggests that, in the presence of the inter-dot tunneling effect, the Fresnel diffraction pattern is contingent on the direction of rotation of the helical phase front of the coupling LG field. Our results offer insights into quantum control of Fresnel diffraction patterns and the identification of OAM in LG beams, presenting potential applications in quantum technologies.

## Introduction

Light diffraction is one of the most interesting phenomena in optics and has attracted major attention since the beginning of optical sciences and has found many applications in optics and other fields of physics such as crystallography. It easily reveals the wave characteristic of the light, especially with the well-known Arago-Poisson-Fresnel spot experiment. The use of diffraction gratings in spectrometry is one of the well-known applications of diffraction. The near field diffraction of the periodic structures or optical gratings that accompany the Talbot effect^1^ or self-imaging phenomenon itself is interesting and has wide applications in lithography^2–4^ and generation of array of vortex beams with multiplication of the incident vortex beam^5^ which can be used for multiple particle rotation in a multi-particle system^6^. In this context, the concept of an electromagnetically induced grating, stemming from electromagnetically induced transparency, was pioneered in 1998^7^ and subsequently demonstrated in cold sodium atoms in 1999^8^. These studies elucidated how leveraging the absorption and dispersion characteristics of electromagnetically induced transparency enables the creation of an atomic grating capable of efficiently diffracting light into the first-order direction.

On the other hand, the diffraction of light beams from structured apertures including gratings having different transmission and reflection profiles, shows many interesting physical effects. Most of the solutions of the wave equation can be generated by imposing the desired boundary conditions through the diffraction of a plane wave or a Gaussian wave. For example, by imposing a cubic phase variation on an incident plane wave, an Airy beam can be simply generated^9–11^. As another new and interesting example, in the diffraction of a plane wave from a radial grating having a sinusoidal or binary profile, a new family of non-diffracting, accelerating, and self-healing beams can be generated^12–14^. In recent decades, a newly applied branch in optics known as diffractive optics has played a very effective role in laser beam shaping^15,16^ and reveals many applications in optical manipulation and optical tweezers. Laser beam shaping with the aid of diffraction provides multi-traps and the orbital rotation of the particles can be simply executed by manually rotating the diffraction element such as a sinusoidal radial grating in its plane around the optical axis^17^. The diffraction of vortex beams from different structured apertures and gratings is also one of the simple ways for characterizing the incident beams^18–23^. The diffraction of a plane wave from a QDM system is also used for the dynamic generation of a periodic intensity pattern with a desired opening number in the near field diffraction region^24^. Here, we are going to investigate the Fresnel diffraction pattern of a weak laser beam passed through a medium including a kind of artificial atoms and introduce a method for dynamic reshaping of the output laser profile.

Quantum coherence and interference play pivotal roles in manipulating the nonlinear optical characteristics of atomic systems^25^. Over the past two decades, a novel class of artificial atoms, known as quantum dots (QDs), has been engineered using semiconductor nanoparticles, offering distinct advantages over natural atoms. Quantum dots demonstrate versatile applications in the domains of quantum optics and quantum information science. Their notable attributes, including substantial nonlinear optical susceptibility, significant electric-dipole moments during intersubband transitions, and exceptional flexibility in device design, underscore their widespread use in these scientific disciplines^26,27^. Furthermore, the interconnection of two or more QDs through inter-dot electron tunneling can give rise to the formation of an artificial molecule termed a QDM^28,29^. The thickness of the potential barrier between adjacent QDs typically falls within the order of a few nanometers. Facilitating the electron tunneling between QDs is achievable by applying a static electric field along the molecular axis^30^. In the context of simulating an atomic vapor cell, a three-dimensional array of QDMs can be generated through a combination of vertical and lateral growth methods for QDMs^31^. The interaction of a homogeneous ensemble of QDMs with applied laser fields results in various optical phenomena, including four-wave mixing generation^32^, entanglement, and quantum-information transfer^33^, optical bistability^34^, transmission and reflection of pulses^35^, as well as control over the Goos–Hänchen shift^36^.

In this manuscript, we investigate the Fresnel diffraction phenomenon exhibited by the probe Gaussian field transmitted through a coherently prepared QDMs. Initially, the solution of the Maxwell and Bloch equations enables us to elucidate the behavior of the output probe field under diverse conditions of the tunneling parameter and the coupling strength of the Laguerre–Gaussian (LG) field. It becomes evident that distinct patterns emerge in the Fresnel diffractions of the output probe field based on these parameters. Considering the coupling field as an LG field introduces a dependency of the Fresnel diffraction patterns of the probe field on the helical phase front of the coupling LG field. Notably, we demonstrate that the presence of the inter-dot tunneling effect, along with the strength, value, and sign of the orbital angular momentum of the coupling LG field, gives rise to various spatial distributions in the Fresnel diffraction patterns of the probe field. These findings highlight the potential for utilizing the OAM of the coupling LG field as a quantum control parameter for manipulating the Fresnel diffraction patterns, offering a straightforward quantum control approach.

## Model and equations

**Figure 1:**
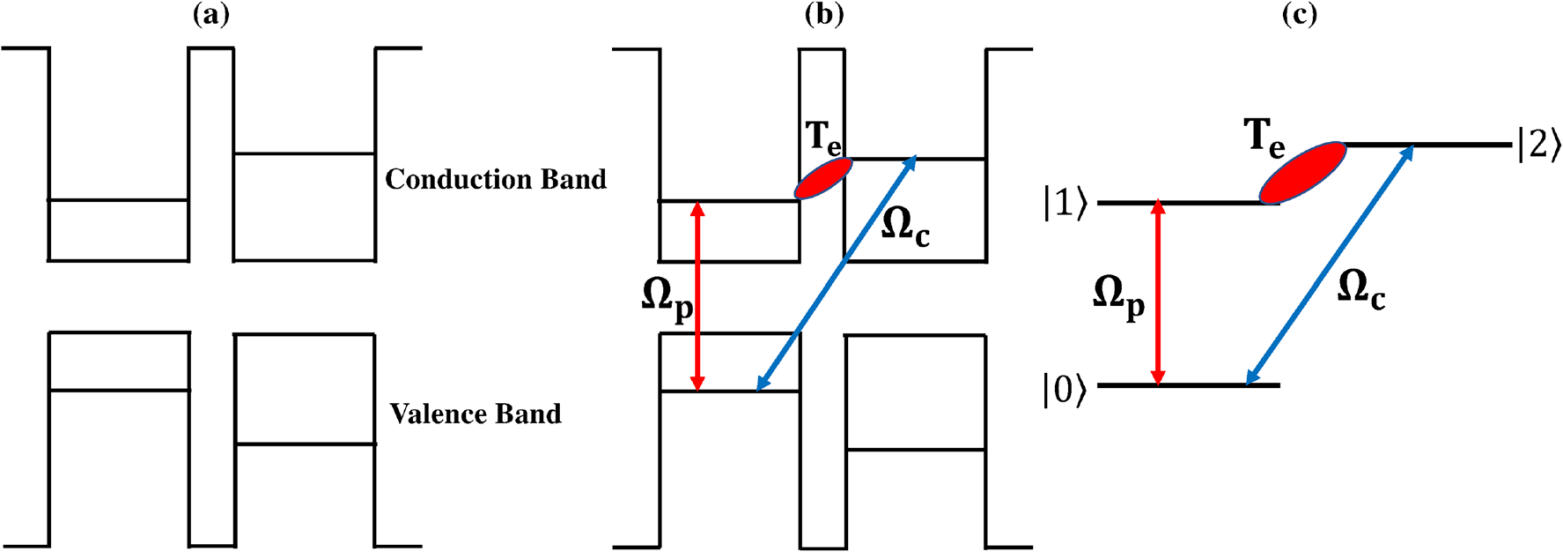
Band diagram of a QDM interacting with probe and coupling fields (**a**) before and (**b**) after applying the gate voltage. (**c**) Schematic diagram of the energy levels QDM system.

We propose a QDM system composed of interconnected pairs of QDs. The application of an external static electric field induces an inter-dot tunneling effect, leading to the establishment of QDMs. For the realization of such a QDM, an asymmetric double-layer InAs/GaAs structure can serve as a practical sample, utilizing self-assembled dot growth technology as a fabrication method^37^. In Fig. 1a, we observe that the conduction bands of the left and right QDs exhibit an energy difference that hinders the formation of a robust coupling between their respective conduction bands. However, the introduction of an external static electric field along the molecular (coupling) axis can potentially enable inter-dot tunneling for the electrons within the conduction bands by eliminating the energy difference between them (see Fig. 1b). Figure 1c illustrates a schematic representation of the three-level *V*-type QDM system following the application of an external electric voltage and in the presence of two applied fields. It is assumed that the $|0\rangle -|1\rangle$ transition is stimulated by a weak probe Gaussian field with a frequency $\omega _{p}$. The Rabi frequency of this field is expressed as:

1 $$\begin{aligned} \Omega _{p}(r):=\frac{\vec {\mu }_{10}\cdot \vec {E}_p}{\hbar }=\Omega _{p0}e^{-r^2/w_G^2}. \end{aligned}$$

Here, $\mu _{10}$, $E_p$, $\hbar$, $w_{G}$, and $\Omega _{p0}$ indicate the induced dipole moment of $|0\rangle -|1\rangle$ transition, electric probe field amplitude, Planck’s constant, waist and constant Rabi frequency of the probe Gaussian field, respectively. It is also considered that the $|0\rangle -|2\rangle$ transition is exited by a strong coupling LG field with the frequency of $\omega _{c}$. The Rabi frequency of the coupling LG field in cylindrical coordinates can be written as

2 $$\begin{aligned} \Omega _{c}(r,\varphi ):=\frac{\vec {\mu }_{20}\cdot \vec {E}_c}{\hbar }=\Omega _{c0}\frac{1}{\sqrt{|l|!}} \left( \frac{\sqrt{2}r}{w_{LG}}\right) ^{|l|}e^{-r^2/w_{LG}^2}e^{il\varphi }, \end{aligned}$$

in which $\mu _{20}$, $E_c$, *l*, $w_{LG}$, $\Omega _{c0}$ stand for the induced dipole moment of $|0\rangle -|2\rangle$ transition, electric coupling field amplitude, OAM value, waist and constant Rabi frequency of the coupling LG field, respectively.

**Figure 2:**
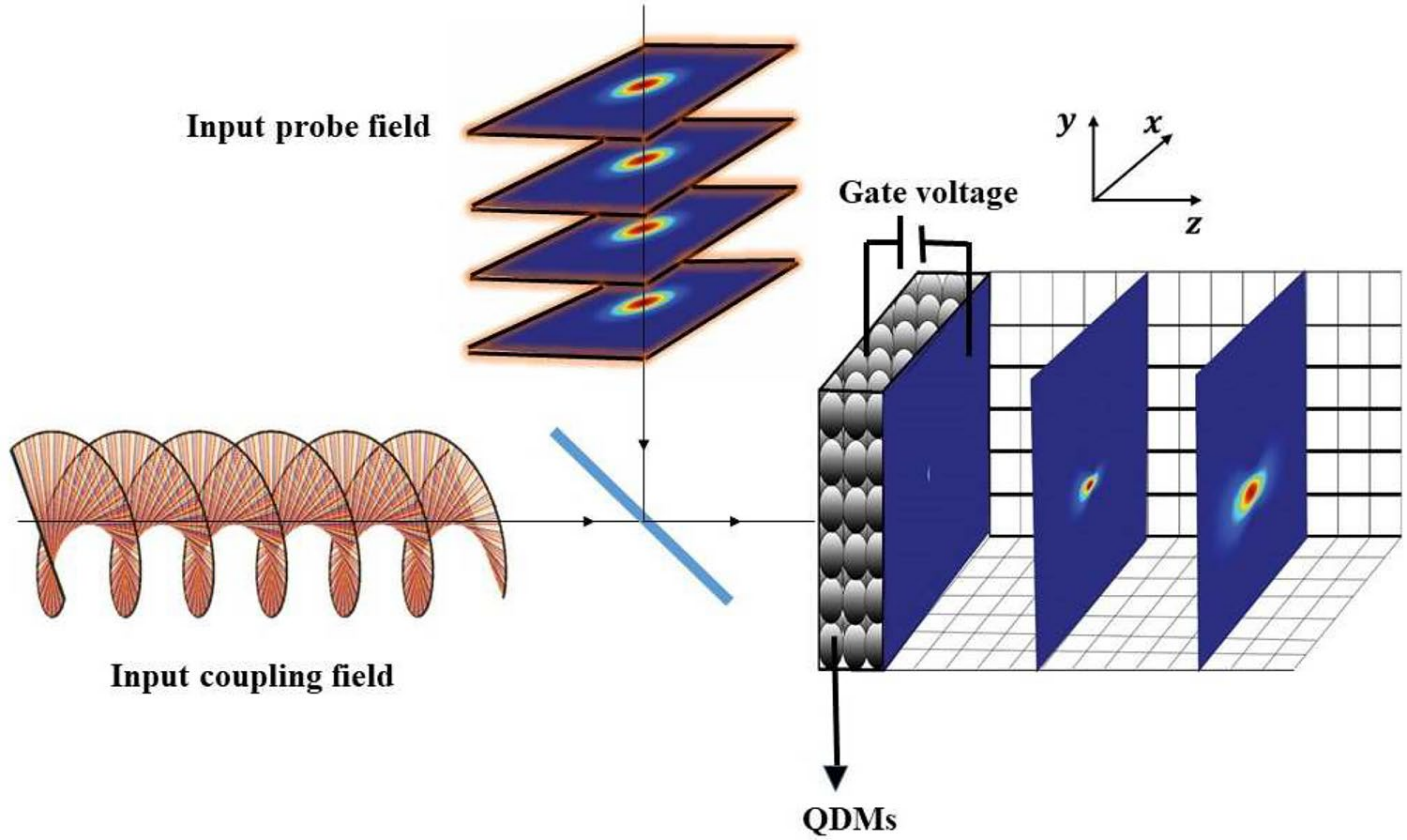
A general schematics of the introduced QDM system interacting with two applied fields and the intensity profiles of the diffracted output probe field.

A general schematic of the introduced QDM system interacting with two applied fields is displayed in Fig. 2. Here, the QDMs presented by spheres are under the external electric voltage. The intensity profiles of the diffracted output probe field are shown at different distances from the exit plane of the QDM medium for a specific set of parameters. It should be noted that the obtained patterns completely depend on the inter-dot tunneling effect and the characteristics of the coupling field. To study the interaction between light and QDM system, one can use the interaction Hamiltonian, under the rotating-wave and dipole approximations, which is given by

3 $$\begin{aligned} H=-\hbar [\Omega _{p}e^{-i(\Delta _{p}+\phi _{p})}|1\rangle \langle 0|+\Omega _{c}e^{-i(\Delta _{c}+\phi _{c})}|2\rangle \langle 0|]+T_{e}e^{-i\omega _{12}t}|2\rangle \langle 1|+C.C., \end{aligned}$$

where the difference frequency between the applied field and corresponding transition, constant phase of the applied field, tunneling strength, and central frequency of $|1\rangle -|2\rangle$ transition are displayed by $\Delta _{p}$($\Delta _c$), $\phi _{p}$($\phi _{c}$), $T_e$, and $\omega _{12}$, respectively. The evolution of the QDM system which interacts with the applied fields can be realized via the von Neumann equation^38^. Therefore, the Bloch equations for the density matrix elements in the presence of an enough strong external electric voltage, $\omega _{12}=0$, are obtained as

4 $$\begin{aligned} {\dot{\rho }}_{00}&= i(\Omega ^{*}_{p}\rho _{10}-\Omega _{p}\rho _{01}-\Omega _{c}e^{i\Delta \phi }\rho _{02}+\Omega _{c}^{*}e^{-i\Delta \phi }\rho _{20}) +\gamma _{10}\rho _{11}+\gamma _{20}\rho _{22},\nonumber \\ {\dot{\rho }}_{11}&= i(\Omega _{p}\rho _{01}-\Omega ^{*}_{p}\rho _{10}+T_{e}(\rho _{12}-\rho _{21}) ) -\gamma _{10}\rho _{11},\nonumber \\ {\dot{\rho }}_{10}&= i(-\Omega _{p}(\rho _{11}-\rho _{00})-\Omega _{c}e^{i\Delta \phi }\rho _{12}-T_{e}\rho _{20}+\Delta _{p}\rho _{10} ) -\Gamma _{10}\rho _{10},\nonumber \\ {\dot{\rho }}_{20}&= i(-\Omega _{c}e^{i\Delta \phi }(\rho _{22}-\rho _{00})-\Omega _{p}\rho _{21}-T_{e}\rho _{10}+\Delta _{c}\rho _{20})-\Gamma _{20}\rho _{20},\nonumber \\ {\dot{\rho }}_{21}&= i(-\Omega ^{*}_{p}\rho _{20}+T_e(\rho _{22}-\rho _{11})+\Omega _{c}e^{i\Delta \phi }\rho _{01}+(\Delta _{c}-\Delta _{p})\rho _{21}) -\Gamma _{21}\rho _{21},\nonumber \\ {\dot{\rho }}_{22}&= -({\dot{\rho }}_{00}+{\dot{\rho }}_{11}). \end{aligned}$$

in which $\gamma _{10}(\gamma _{20})$, $\Gamma _{ij}$, and $\Delta \phi =\phi _p-\phi _c$ are the spontaneous decay rate from the upper level $|1\rangle$($|2\rangle$) to the lower one $|0\rangle$, dephasing rate, and relative phase of the applied fields, respectively. Now, we analytically solve Eq. (4) for $\Gamma _{20}=\gamma _{20}=0$ and $\Delta _p=\Delta _c=0$ in the steady state and obtain the coherence term, $\rho _{10}$, as

5 $$\begin{aligned} \rho _{10}=\frac{i\gamma _{10}\Gamma _{21}|\Omega _{c}|^{2}\Omega _{p} +2\gamma _{10}T_{e}\Omega _ce^{i\Delta \phi }(T_e^2-|\Omega _{c}|^{2})}{A}, \end{aligned}$$

in which

$$\begin{aligned} A&= 2\gamma _{10}T_e^4+6T_e^2|\Omega _{c}|^{2}(2\Gamma _{10}-\gamma _{10}) +4\gamma _{10}|\Omega _{c}|^{2}(\Gamma _{10}\Gamma _{21}+|\Omega _{c}|^{2}). \end{aligned}$$

It is worth noting that the first term of $\rho _{10}$ is the direct response of the QDM medium to the probe field, while, the second one originates from the scattering of the coupling field into the probe field via inter-dot tunneling effect. The inter-dot tunneling effect enables the generation of a linear superposition of the electric field amplitudes of two probe and coupling fields, each making distinct contributions. On the other hand, the susceptibility of the QDM medium which is proportional to the coherence term, $\chi =2N |\mu _{10}|^{2}\rho _{10}/\hslash \varepsilon _{0}\Omega _{p}$, can be used for obtaining the output probe field via the Maxwell wave equation. Here, the density of the QDMs number is indicated by *N*.

The Maxwell wave equation in the slowly varying envelope approximation, as $\partial E_{p}/\partial Z =ik_p\chi E_{p}$, helps us to study the evolution of the probe field passing through the QDM system along the propagation direction of *z*-axis. Here, the wave number of the probe field is denoted by $k_p$. It is assumed that the diffraction of the probe field inside the QDM medium is negligible. Considering the probe field as a Gaussian field [Eq. (1)], The output probe field at the exit plane of the QDM medium, $z=L$, is obtained as

6 $$\begin{aligned} E_{p}(r,\varphi ,z=L)=E_{p0}e^{-r^2/w_{G}^{2}}exp\{\alpha [-Im(\gamma \rho _{10}/\Omega _{p})+iRe(\gamma \rho _{10}/\Omega _{p})]\}, \end{aligned}$$

where $\gamma$ is a decay rate scaling and the dimensionless parameter $\alpha =N |\mu _{10}|^{2}k_p L/\hslash \varepsilon _{0}\gamma$ is a scale of medium absorption for the probe field. In this case, one can suppose that the strong coupling field experiences no change as propagating through the QDM medium. The diffraction pattern of the output probe field can be calculated by the Fresnel diffraction integral

7 $$\begin{aligned} E'_{p}(x,y,z)=\frac{e^{ik_{p}z}}{i\lambda _{p}z}\int _{0}^{2\pi }\int _{0}^{\infty }rE_{p}(r,\varphi ,z=L) e^{ik_{p}(x-r cos\varphi )^{2}/2z}e^{ik_{p}(y-r sin\varphi )^{2}/2z} dr d\varphi , \end{aligned}$$

in which $\lambda _{p}$, *z*, (*x*, *y*), and $(r,\varphi )$ describe the wavelength of the probe field, distance of the observation plane from the output plane along the propagation axis, coordinates of the observation plane, and the output one, respectively. The unit of measurement for the z-axis in our surveys is meters. Substituting Eq. (6) into Eq. (7), the magnitude and phase of the diffracted output probe field, $E'_{p}(x,y,z)$, can be numerically obtained in different distances from the exit plane of the QDM medium.

## Results and discussions

In this investigation, we explore the influence of the inter-dot tunneling effect and the strength of the coupling LG field on the propagation of the probe Gaussian field within the QDM medium, utilizing the Eq. (6). The electric component of the output probe field significantly plays a major role in the determination of the two-dimensional Fresnel diffraction pattern^39^.

**Figure 3:**
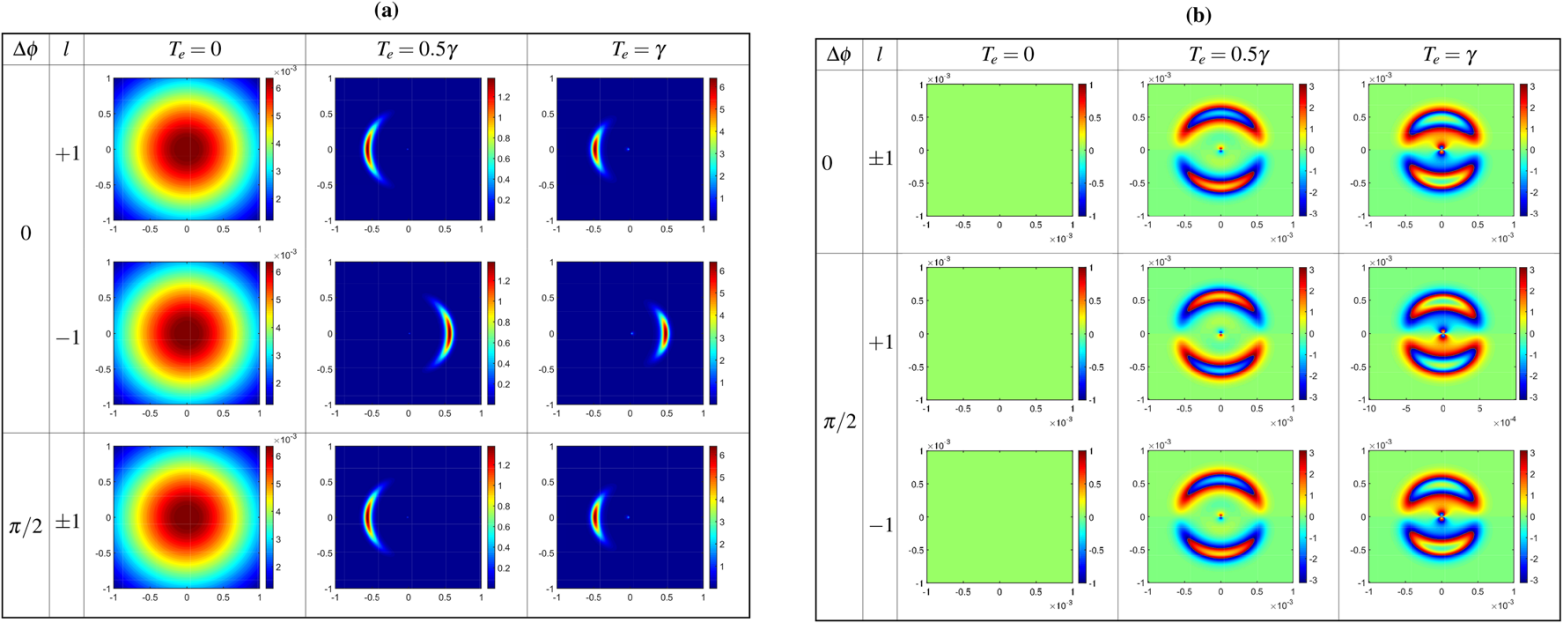
Output probe amplitude (**a**), and phase (**b**) profiles at the exit plane of the medium, $Z=L$, as a function of *x* and *y* for different values of the tunneling parameter and two opposite modes of the coupling LG field, i.e. $l=1$ and $-1$. The horizontal, *x*, and vertical, *y*, axes are taken in *mm*. Used parameters related to the QDM system are considered to be $\Gamma _{20}=\gamma _{20}=0$, $\gamma _{10}=0.554\gamma$, $\Gamma _{10}=5.54\gamma$, $\Gamma _{12}=2\gamma$, $\gamma =1meV$^40^, and $\alpha =10$. The applied fields characteristics are chosen to be $w_{G}=1.1mm$, $\Omega _{p0}=0.1\gamma$, $E_{p0}=0.01$, $w_{LG}=270$ μm, and $\Omega _{c0}=\gamma$, under two-photon resonance condition $\Delta _p=\Delta _c=0$.

In this regards, Fig. 3 displays the output probe amplitude (a) and phase (b) profiles for $\Delta \phi =0, \pi /2$ at the exit plane of the medium ($z=L$), as functions of *x* and *y*, for various values of the tunneling parameter and two opposing modes of the coupling LG field, i.e., $l=1$ and $-1$. The horizontal (*x*) and vertical (*y*) axes are measured in millimeters. The parameters associated with the QDM system are specified as follows: $\Gamma _{20}=\gamma _{20}=0$, $\gamma _{10}=0.554\gamma$, $\Gamma _{10}=5.54\gamma$, $\Gamma _{12}=2\gamma$, where $\gamma =1$ meV^40^, and $\alpha =10$. The characteristics of the applied fields are selected as $w_{G}=1.1$ mm, $\Omega _{p0}=0.1\gamma$, $E_{p0}=0.01$, $w_{LG}=270$ μm, and $\Omega _{c0}=\gamma$, under the two-photon resonance condition ($\Delta _p=\Delta _c=0$). As depicted in the left column of Fig. 3a, the output probe amplitude profile exhibits a Gaussian function shape in the absence of inter-dot tunneling effects. In this scenario, the OAM of the coupling LG field does not influence the output probe field. However, the presence of inter-dot tunneling alters the amplitude profile of the output probe field, contingent upon the sign of the topological charge of the coupling LG beam, particularly evident for $\Delta \phi =0$ as illustrated in the middle and right columns of Fig. 3a. Figure 3b elucidates the relation between the phase profile of the output probe field and the helical wavefront of the coupling LG field. It is evident that the phase profile is contingent on the sign of the coupling LG field’s OAM only when the tunneling parameter is non-zero, specifically for $\Delta \phi =\pi /2$ as demonstrated in the middle and right columns of Fig. 3b. Consequently, the characteristics of the output probe field can be manipulated by tunneling parameters through the establishment of a closed-loop QDM system. The phase-dependent behavior of the amplitude and phase profiles in the presence of inter-dot tunneling can be elucidated by the analytical result provided by Eq. (5). It is noteworthy that in the calculation of the diffraction patterns of the output probe field, the dominant role is played by the imaginary part. However, the deflection of the Fresnel diffraction pattern is determined by the real part of the coherence term. With $\Delta \phi = \pi /2$, the real part of the coupling Rabi frequency corresponds to $\sin (l\phi )$. Consequently, the real part of the coherence term becomes odd with respect to *l*, and the position of the Fresnel diffraction pattern depends on the sign of the topological charge. Selecting $\Delta \phi = \pi /2$ is anticipated to yield a diffraction pattern contingent on both the magnitude and sign of the topological charge of the coupling field.

**Figure 4:**
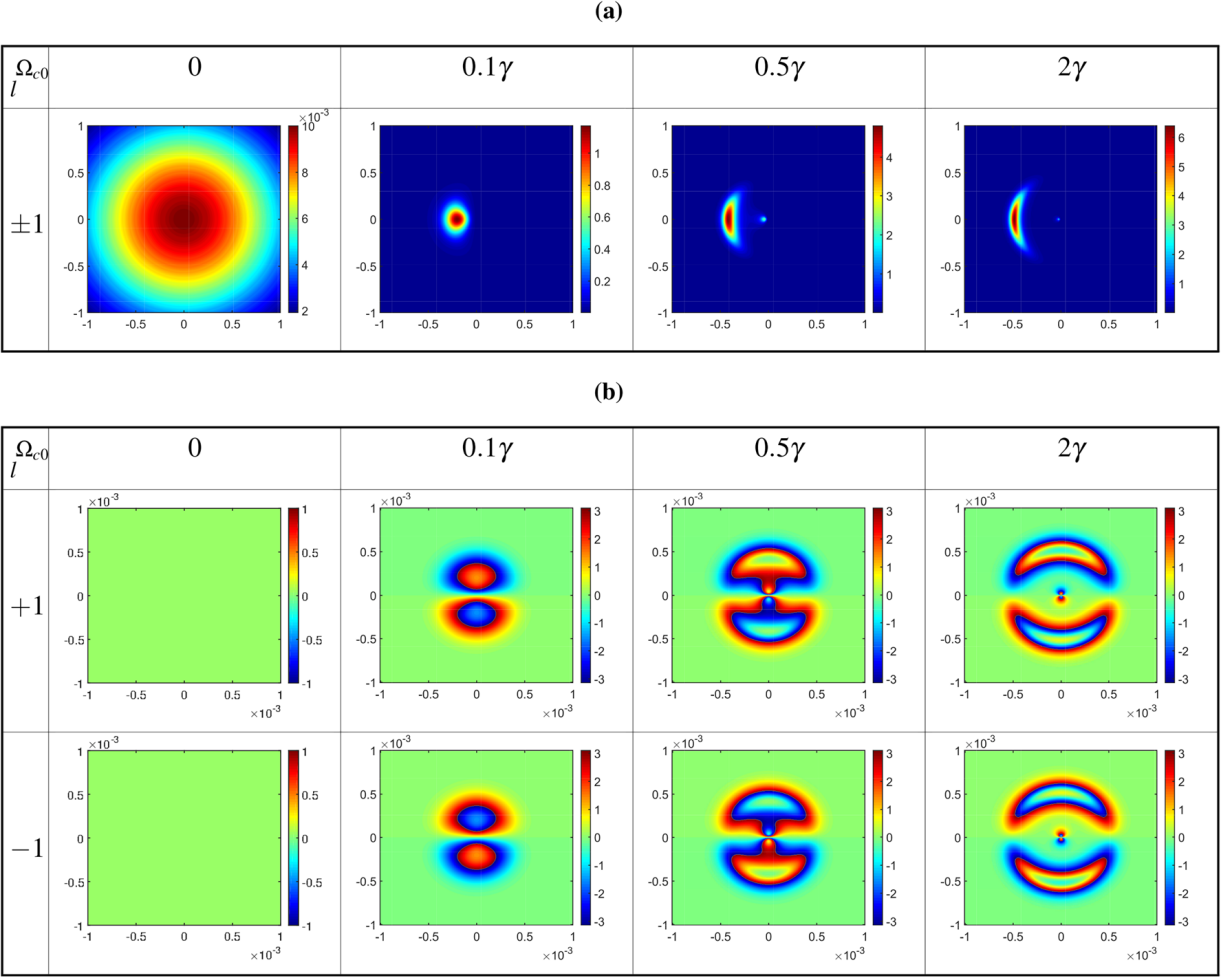
Output probe amplitude (**a**) and phase (**b**) profiles at the exit plane of the medium versus *x* and *y* for different values of the constant Rabi frequency of coupling LG field and $T_e=\gamma$ under the same parameters of Fig. 3.

Figure 4 presents the output probe amplitude (a) and phase (b) profiles for $\Delta \phi =\pi /2$ at the exit plane of the QDM medium versus *x* and *y* for different values of the constant Rabi frequency of coupling LG field and $T_e=\gamma$ under the same parameters of Fig. 3. An investigation of Figs. 3 and 4 indicate that the effect of the strength of the coupling LG field on the output probe field is similar to the tunneling parameter. The output probe field has still a Gaussian form if the coupling LG field is turned off. However, in the presence of the coupling LG field, the output probe field amplitude is concentrated at a region and its phase profile exits the planar mode. This result originates from the establishment of a closed-loop quantum system by applying an external gate voltage as well as coupling the LG field; so that, in the presence of these external factors, the phase profile of the output probe field has an explicit dependency on the sign of the OAM of the coupling LG field. In the following, we study the Fresnel diffraction of the output probe field in free space by numerical solving of Eq. (7).

**Figure 5:**
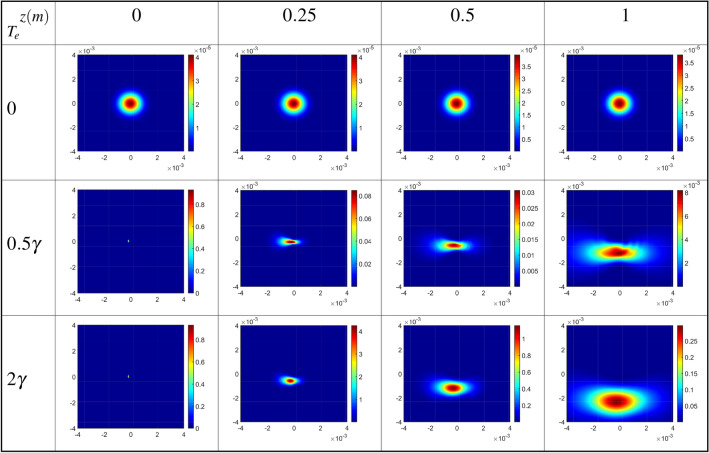
Diffraction patterns of the output probe field as a function of *x* and *y* in the absence ($T_e=0$), first row, and in the presence of the inter-dot tunneling effect ($T_e=0.5\gamma$), second row, and ($T_e=2\gamma$), third row, for positive ($l=1$) OAM values of the coupling LG field at different distances from the exit plane of the QDM medium. The wavelength of the probe field is fixed at $\lambda _{p}=870nm$. Other parameters are the same as in Fig. 3.

In Fig. 5, the diffraction patterns of the output probe field are plotted as a function of *x* and *y* in the absence ($T_e=0$), first row, and in the presence of the inter-dot tunneling effect ($T_e=0.5\gamma$), second row, and ($T_e=2\gamma$), third row, for positive ($l=1$) OAM values of the coupling LG field at different distances from the exit plane of the QDM medium. The wavelength of the probe field is fixed at $\lambda _{p}=870nm$. Other parameters are the same as in Fig. 3. The first row of Fig. 5 shows the Fresnel diffraction patterns of the output probe Gaussian field. It should be mentioned that the output probe field profile, in the absence of the inter-dot tunneling effect, remains as a Gaussian one with some ignorable changes in the value of intensity, during propagating in free space. In the second row of Fig. 5, the size of the diffraction pattern increases for far away distances. It is figured out that the resulting diffraction patterns can be used as an accurate method for identification of the wavefront of an LG field just in the presence of the inter-dot tunneling effect. It can be found that the Gaussian-like diffraction pattern turns into a bow by turning on the gate voltage, i.e. in the presence of the inter-dot tunneling parameter. Afterward, enhancement of the tunneling parameter leads to a clear shift in the tunneling-induced diffraction pattern. It means that the diffraction can be controlled by a tunable parameter of the QDM medium, $T_e$.

**Figure 6:**
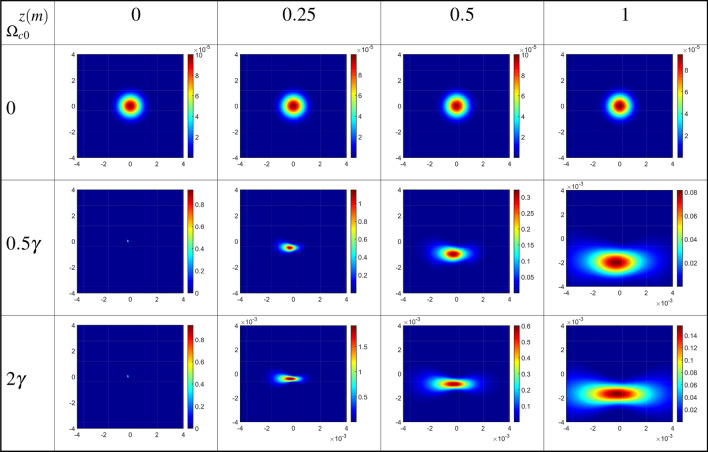
Diffraction patterns of the output probe field as a function of *x* and *y* for different values of the constant Rabi frequency of coupling LG field, as the first LG mode $l=1$, and $T_e=\gamma$ at different distances from the exit plane of the QDM medium. Other used parameters are the same as in Fig. 5.

Now, we obtain the diffraction patterns of the output probe field as a function of *x* and *y* for different values of the constant Rabi frequency of coupling LG field, as the first LG mode $l=1$, and $T_e=\gamma$ at different distances from the exit plane of the QDM medium, in Fig. 6. Other used parameters are the same as in Fig. 5. In studying the effect of the coupling field’s characteristics on the Fresnel diffraction of the output probe field, it should be noted that the presence of the inter-dot tunneling effect is also very important. The results of the first row of Fig. 6 in the absence of the coupling LG field are similar to those of the zero tunneling parameters in the first row of Fig. 5. Moreover, the spatial distribution of the Fresnel diffraction patterns can change by increasing the strength of the coupling LG field. It is notable that the diffraction efficiency, quantified as the ratio of the power of the output diffracted probe field to the input field, exceeds unity in the diffraction of light through quantum dot molecules. This phenomenon arises from the scattering of energy from the coupling field into the output probe field via inter-dot tunneling, elucidated by the second term of Eq. (5).

**Figure 7:**
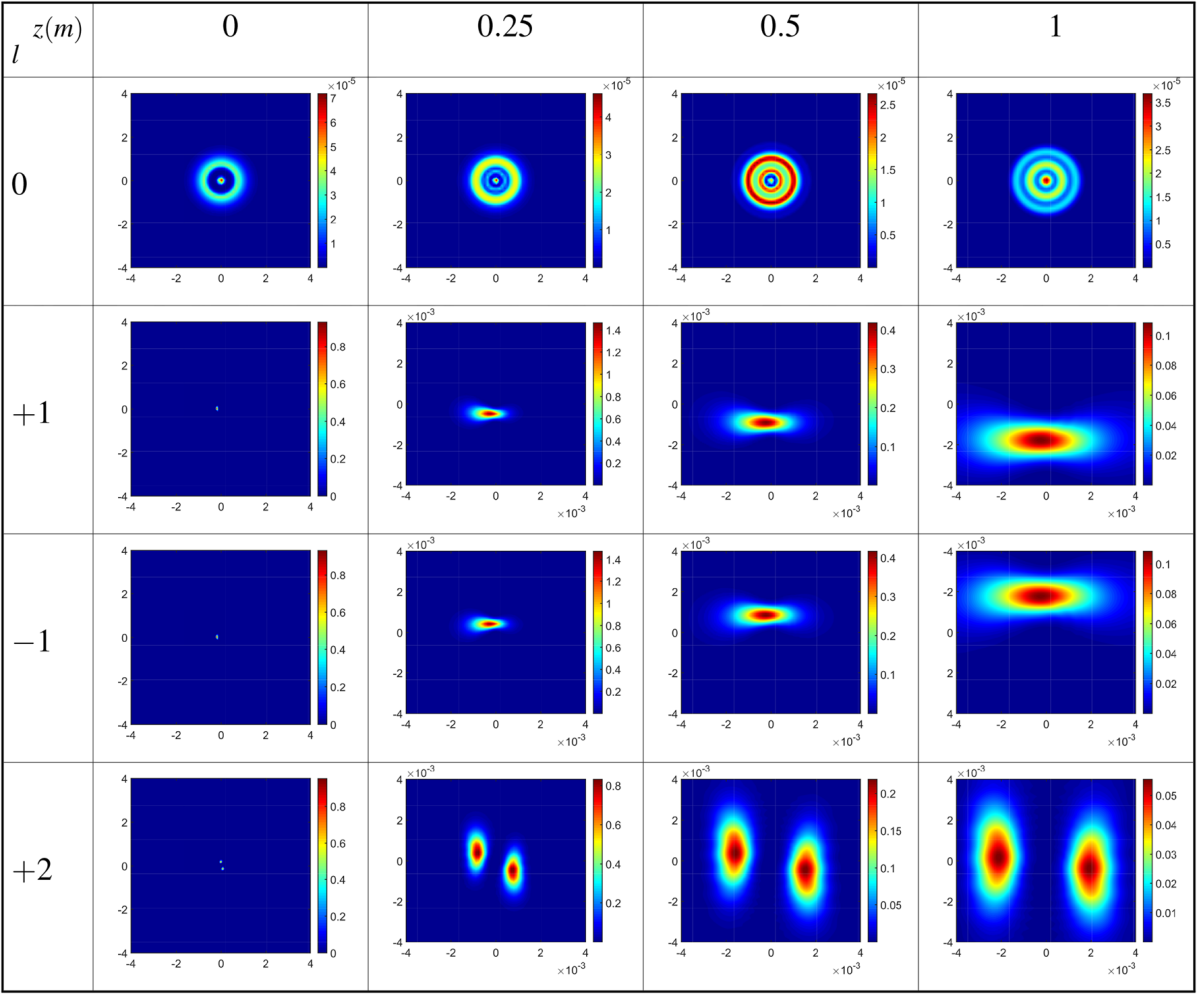
Diffraction patterns of the output probe field versus *x* and *y* for different OAM values of the coupling LG field at different distances from the exit plane of the QDM medium. The value of tunneling parameter is considered to be $T_e=\gamma$ under the same parameters of Fig. 5.

Now, we are going to explore how the value of the OAM of the coupling LG field can affect the output probe field and its diffraction patterns in the presence of the inter-dot tunneling effect. The diffraction patterns of the output probe field are plotted versus *x* and *y* for different OAM values of the coupling LG field at different distances from the exit plane of the QDM medium, in Fig. 7. The value of the tunneling parameter is considered to be $T_e=\gamma$ under the same parameters of Fig. 5. In this situation, a closed-loop QDM system has been established by applying the external gate voltage, coupling, and probe fields. As exhibited in the first row of Fig. 7, by passing the output probe field, which resulted from the interference between two Gaussian fields with different waists, more maximum intensity rings have appeared in its intensity profiles at different distances from the exit plane of the QDM medium. The obtained results for the first mode of the coupling LG field, for positive ($l=1$), in the second row, and negative ($l=-1$), in the third row, indicate that the diffraction pattern tends to a special direction and spreads by propagation along *z*-direction. However, the diffraction patterns are divided into two parts when the coupling LG field is considered in its second mode, $l=2$, see the fourth row of Fig. 7. So, the diffraction pattern of the output probe field can be manipulated by the magnitude and sign of the OAM of the coupling LG field whenever an external gate voltage is applied to the QDM medium. Our numerical findings reveal that by employing a linear superposition of two Laguerre–Gaussian (LG) modes with opposite helical wavefronts for the coupling field^41,42^, a more intricate Fresnel diffraction pattern devoid of deflection emerges. It is noteworthy that the deflections of the two opposite helical LG beams occur in opposing directions. Consequently, the deflection of the Fresnel diffraction pattern for $l=1$ is counteracted by the contribution of $l=-1$. In essence, through the consideration of a linear superposition of two LG modes with opposite helical wavefronts, the term $\cos (l\phi )$ emerges in the coupling Rabi frequency. As a result, the real part of the coherence term becomes even with respect to *l*, rendering deflection unattainable in the Fresnel diffraction pattern.

The discovered results hold potential for identifying the Orbital Angular Momentum (OAM) of electromagnetic fields, a pivotal aspect with applications spanning communication technology advancement and the elucidation of fundamental physical principles. Furthermore, these findings offer promise in optical switching applications, wherein the modulation of diffraction pattern intensity through external voltage application to the sample facilitates control over optical signal transmission.

## Conclusion

We have studied the effect of the external electric voltage on the Fresnel diffraction of the output probe field in a QDM medium. It has been shown that the strength of the inter-dot tunneling, the coupling field intensity and phase profiles have an important role in the propagation of the probe field out of the QDM medium. The inter-dot tunneling effect, which is possible in the presence of the external gate voltage, provides an occasion for changing the diffraction pattern profiles via the coupling LG field. It is intriguing to note that the diffraction pattern of the output probe field completely depends on the direction of rotation of the helical phase front of the coupling LG field in the presence of the inter-dot tunneling effect. Our results are physically rooted in the interference of the applied fields which is due to the inter-dot tunneling. It would be valuable to find some applications of our reported results in the manipulation of the Fresnel diffraction patterns via simple adjusting of the external voltage.

## Data Availability

The datasets used and/or analysed during the current study are available from the corresponding author on reasonable request.

## References

[1] Talbot HF (1836). Facts relating to optical science. Philos. Mag..

[2] Alkaisi MM, Blaikie RJ, McNab SJ, Cheung R, Cumming DRS (1999). Sub-diffraction-limited patterning using evanescent near-field optical lithography. Appl. Phys. Lett..

[3] Naqavi A, Peter Herzig H, Rossi M (2016). High-contrast self-imaging with ordered optical elements. J. Opt. Soc. Am. B.

[4] Rasouli S, Hebri D (2017). Contrast-enhanced quarter-Talbot images. J. Opt. Soc. Am. A.

[5] Rasouli S, Hebri D (2019). Theory of diffraction of vortex beams from 2D orthogonal periodic structures and Talbot self-healing under vortex beam illumination. J. Opt. Soc. Am. A.

[6] Ikonnikov DA, Myslivets SA, Volochaev MN, Arkhipkin VG, Vyunishev AM (2020). Two-dimensional Talbot effect of the optical vortices and their spatial evolution. Sci. Rep..

[7] Ling HY, Li YQ, Xiao M (1998). Electromagnetically induced grating: Homogeneously broadened medium. Phys. Rev. A.

[8] Mitsunaga M, Imoto N (1999). Observation of an electromagnetically induced grating in cold sodium atoms. Phys. Rev. A.

[9] Siviloglou GA, Broky J, Dogariu A, Christodoulides DN (2007). Observation of accelerating airy beams. Phys. Rev. Lett..

[10] Siviloglou GA, Christodoulides DN (2007). Accelerating finite energy Airy beams. Opt. Lett..

[11] Abdollahpour D, Lotfollahi Sheikhdarabad M, Yeganeh M, Rasouli S (2019). Generation and characterization of adjustable pure third-order spatial phase by tuning optical aberrations. J. Opt..

[12] Rasouli S, Khazaei AM, Hebri D (2018). Talbot carpet at the transverse plane produced in the diffraction of plane wave from amplitude radial gratings. J. Opt. Soc. Am. A.

[13] Rasouli S, Khazaei AM, Hebri D (2018). Radial carpet beams: A new class of nondiffracting, accelerating, and self-healing beams. Phys. Rev. A.

[14] Hebri D, Rasouli S (2018). Combined half-integer Bessel-like beams: A set of solutions of the wave equation. Phys. Rev. A.

[15] Tricoles G (1987). Computer generated holograms: An historical review. Appl. Opt..

[16] Rasouli S, Khazaei AM (2019). An azimuthally-modified linear phase grating: Generation of varied radial carpet beams over different diffraction orders with controlled intensity sharing among the generated beams. Sci. Rep..

[17] Bayat J, Hajizadeh F, Khazaei AM, Rasouli S (2020). Gear-like rotatable optical trapping with radial carpet beams. Sci. Rep..

[18] Guo CS, Lu LL, Wang HT (2009). Characterizing topological charge of optical vortices by using an annular aperture. Opt. Lett..

[19] Moreno I, Davis JA, Pascoguin BML, Mitry MJ, Cottrell DM (2009). Vortex sensing diffraction gratings. Opt. Lett..

[20] Hebri D, Rasouli S, Yeganeh M (2018). Intensity-based measuring of the topological charge alteration by the diffraction of vortex beams from amplitude sinusoidal radial gratings. J. Opt. Soc. Am. B.

[21] Dai K, Gao C, Zhong L, Na Q, Wang Q (2015). Measuring OAM states of light beams with gradually-changing-period gratings. Opt. Lett..

[22] Hebri D, Rasouli S, Dezfouli AM (2019). Theory of diffraction of vortex beams from structured apertures and generation of elegant elliptical vortex Hermite-Gaussian beams. J. Opt. Soc. Am. A.

[23] Amiri P, Dezfouli AM, Rasouli S (2020). Efficient characterization of optical vortices via diffraction from parabolic-line linear gratings. J. Opt. Soc. Am. B.

[24] Azizi B, Amini Sabegh Z, Mahmoudi M, Rasouli S (2021). Tunneling-induced Talbot effect. Sci. Rep..

[25] Ficek Z, Swain S (2004). Quantum Coherence and Interference; Theory and Experiments.

[26] Oosterkamp H, Fujisawa T, Van Der Wiel WG, Ishibashi K, Hijman RV, Tarucha S (1998). Microwave spectroscopy of a quantum-dot molecule. Nature (London).

[27] Zrenner A, Beham E, Stufler S, Findeis F, Bichler M, Abstreiter G (2002). Coherent properties of a two-level system based on a quantum-dot photodiode. Nature (London).

[28] Villas-Bôas JM, Govorov AO, Ulloa SE (2004). Coherent control of tunneling in a quantum dot molecule. Phys. Rev. B.

[29] Müller K, Bechtold A, Ruppert C, Zecherle M, Reithmaier G, Bichler M, Krenner HJ, Abstreiter G, Holleitner AW, Villas-Bôas JM, Betz M, Finley JJ (2012). Electrical control of interdot electron tunneling in a double InGaAs quantum-dot nanostructure. Phys. Rev. Lett..

[30] Beirne GJ, Hermannstädter C, Wang L, Rastelli A, Müller E, Schmidt OG, Michler P (2007). Tunable lateral tunnel coupling between two self-assembled InGaAs quantum dots. Proc. SPIE.

[31] Yakes MK, Cress CD, Tischler JG, Bracker AS (2010). Three-dimensional control of self-assembled quantum dot configurations. ACS Nano.

[32] Sitek A, Machnikowski P (2009). Four-wave mixing optical response of an ensemble of quantum dot molecules. Phys. Status Solidi C.

[33] Lü XY, Wu J, Zheng LL, Zhan ZM (2011). Voltage-controlled entanglement and quantum-information transfer between spatially separated quantum-dot molecules. Phys. Rev. A.

[34] Vafafard A, Goharshenasan S, Nozari N, Mortezapour A, Mahmoudi M (2013). Phase-dependent optical bistability in the quantum dot nanostructure molecules via inter-dot tunneling. J. Lumin..

[35] Nasehi R, Mahmoudi M, Sahrai M (2016). Phase-dependent optical bistability in the quantum dot nanostructure molecules via inter-dot tunneling. Laser Phys..

[36] Nasehi R, Asadpour SH, Rahimpour Soleimani H, Mahmoudi M (2016). Controlling the Goos–Hänchen shift via incoherent pumping field and electron tunneling in the triple coupled InGaAs/GaAs quantum dots. Chin. Phys. Lett..

[37] Beirne GJ, Hermannstädter C, Wang L, Rastelli A, Schmidt OG, Michler P (2006). Quantum light emission of two lateral tunnel-coupled (In, Ga) As/GaAs quantum dots controlled by a tunable static electric field. Phys. Rev. Lett..

[38] Breuer HP, Petruccione F (2002). The Theory of Open Quantum Systems.

[39] Wen J, Du S, Chen H, Xiao M (2011). Electromagnetically induced Talbot effect. Appl. Phys. Lett..

[40] Li J, Yu R, Liu J, Huang P, Yang X (2008). Voltage-controlled optical bistability of a tunable three-level system in a quantum-dot molecule. Phys. E..

[41] Rasouli S, Amiri P, Kotlyar VV, Kovalev AA (2021). Characterization of a pair of superposed vortex beams having different winding numbers via diffraction from a quadratic curved-line grating. JOSA B.

[42] Kotlyar VV, Kovalev AA, Amiri P, Soltani P, Rasouli S (2021). Topological charge of two parallel Laguerre–Gaussian beams. Opt. Express.

